# The RNA binding protein MEX3A promotes tumor progression of breast cancer by post-transcriptional regulation of IGFBP4

**DOI:** 10.1007/s10549-023-07028-5

**Published:** 2023-07-11

**Authors:** Wenhu Chen, Liqiang Hu, Xuemei Lu, Xiaofei Wang, Changan Zhao, Chen Guo, Xiaoyan Li, Yuqin Ding, Hongguang Zhao, Dongdong Tong, Lifang Wang, Chen Huang

**Affiliations:** 1 Department of Cell Biology and Genetics, School of Basic Medical Sciences, Xi’an Jiaotong University Health Science Center, No. 76 Yanta West Road, Xi’an, 710061 Shanxi China; 2 School of Basic Medical Sciences & Forensic Medicine, Hangzhou Medical College, Hangzhou, 310053 China; 3 Cancer Institute of Integrated Traditional Chinese and Western Medicine, Zhejiang Academy of Traditional Chinese Medicine, Hangzhou, 310012 China; 4 College of Pharmaceutical Sciences, Zhejiang Chinese Medical University, Hangzhou, 310053 China; 5 Biomedical Experimental Center of Xi’an Jiaotong University, Xi’an, 710061 China; 6 Department of Pathology, School of Basic Medical Sciences, Xi’an Jiaotong University Health Science Center, Xi’an, 710061 China; 7 Department of Breast Surgery, Cancer Hospital of the University of Chinese Academy of Sciences (Zhejiang Cancer Hospital), Hangzhou, 310005 China; 8 Department of Thoracic Surgery, Cancer Hospital of the University of Chinese Academy of Sciences (Zhejiang Cancer Hospital), Hangzhou, 310005 China; 9 Key Laboratory of Environment and Genes Related to Diseases, Xi’an Jiaotong University Health Science Center, Xi’an, 710061 China; 10 College of Innovation & Entrepreneurship, Hangzhou Medical College, No. 548 Binwen Road, Hangzhou, 310053 Zhejiang China; 11 Key Laboratory of Shaanxi Province for Craniofacial Precision Medicine Research, College of Stomatology, Xi’an Jiaotong University, Xi’an, 710061 China

**Keywords:** MEX3A, Breast cancer, RNA-binding protein, IGFBP4

## Abstract

**Purpose:**

Breast cancer (BC) is the most frequent malignant tumor in women worldwide with exceptionally high morbidity. The RNA-binding protein MEX3A plays a crucial role in genesis and progression of multiple cancers. We attempted to explore its clinicopathological and functional significance in BC in which MEX3A is expressed.

**Methods:**

The expression of MEX3A detected by RT-qPCR and correlated the results with clinicopathological variables in 53 BC patients. MEX3A and IGFBP4 profile data of BC patients were downloaded from TCGA and GEO database. Kaplan-Meier (KM) analysis was used to estimate the survival rate of BC patients. Western Blot, CCK-8, EdU, colony formation and flow cytometry were performed to investigate the role of MEX3A and IGFBP4 in BC cell proliferation, invasion and cell cycle in vitro. A subcutaneous tumor mouse model was constructed to analyze in vivo growth of BC cells after MEX3A knockdown. The interactions among MEX3A and IGFBP4 were measured by RNA pull-down and RNA immunoprecipitation.

**Results:**

The expression of MEX3A was upregulated in BC tissues compared to adjacent tissues and high expression of MEX3A was associated with poor prognosis. Subsequent in vitro studies demonstrated that MEX3A knockdown inhibited BC cells proliferation and migration, as well as xenograft tumor growth in vivo. The expression of IGFBP4 was significantly negatively correlated with MEX3A in BC tissues. Mechanistic investigation showed that MEX3A binds to IGFBP4 mRNA in BC cells, decreasing IGFBP4 mRNA levels, which further activated the PI3K/AKT and other downstream signaling pathways implicated cell cycle progression and cell migration.

**Conclusion:**

Our results indicate that MEX3A plays a prominent oncogenic role in BC tumorigenesis and progression by targeting IGFBP4 mRNA and activating PI3K/AKT signaling, which can be used as a novel therapeutic target for BC.

**Supplementary Information:**

The online version contains supplementary material available at 10.1007/s10549-023-07028-5.

## Introduction

Breast cancer (BC) is the most common malignant tumor among women and is the major cause of most cancer-related deaths in the world [1, 2]. Despite tremendous efforts to improve early diagnosis and new therapeutic strategy, BC remains a major health problem and face unsatisfactory prognosis, especially in patients with advanced disease [3]. Thus, it is worth to discover the potential mechanism of BC pathogenesis and find new therapeutic targets.

MEX3A belongs to the MEX3 family of evolutionarily conserved RNA-binding protein (RBP) in mammals (MEX3A-D) [4]. The human MEX3s are phosphoproteins that consist of two N-terminal K homology (KH) domains and one C-terminal RING finger module [5]. MEX3s bind target RNAs through their KH domains and controls mRNA post-transcriptional gene regulation, with implications regarding embryonic development, epithelial homeostasis and carcinogenesis [6]. Previous studies have shown that MEX3A is one novel components of processing bodies (P bodies), which are sites of mRNA decay and storage of untranslated transcripts, implicating that MEX3A may be involved in the regulation of a few mRNA decays [7]. Recently, the dysregulation of MEX3A has been described in several types of human cancers, such as gastric carcinoma [8], glioma [9] and colorectal cancer [10]. However, the role and deep-going regulatory mechanism of MEX3A in breast cancer still require further elucidation.

In the present study, we analyzed the expression of MEX3A of BC tissues from the TCGA and GEO data. Meanwhile, we identified that MEX3A was markedly up-regulated in BC tissues and cells. These results illuminated that MEX3A may act as a promoter in tumor progression. In addition, the interaction of MEX3A and IGFBP4 mRNA was confirmed. Knockdown of MEX3A significantly inhibited breast cancer cell proliferation, migration, and invasion by promoting IGFBP4 expression, revealing the regulatory mechanism of MEX3A targeting IGFBP4 in BC cells. A further exploring the molecular mechanism of MEX3A in BC progression might open promising therapeutic strategy against BC development.

## Results

### MEX3A is highly expressed in BC tissues and cell lines and predicts poor prognosis

**Fig. 1:**
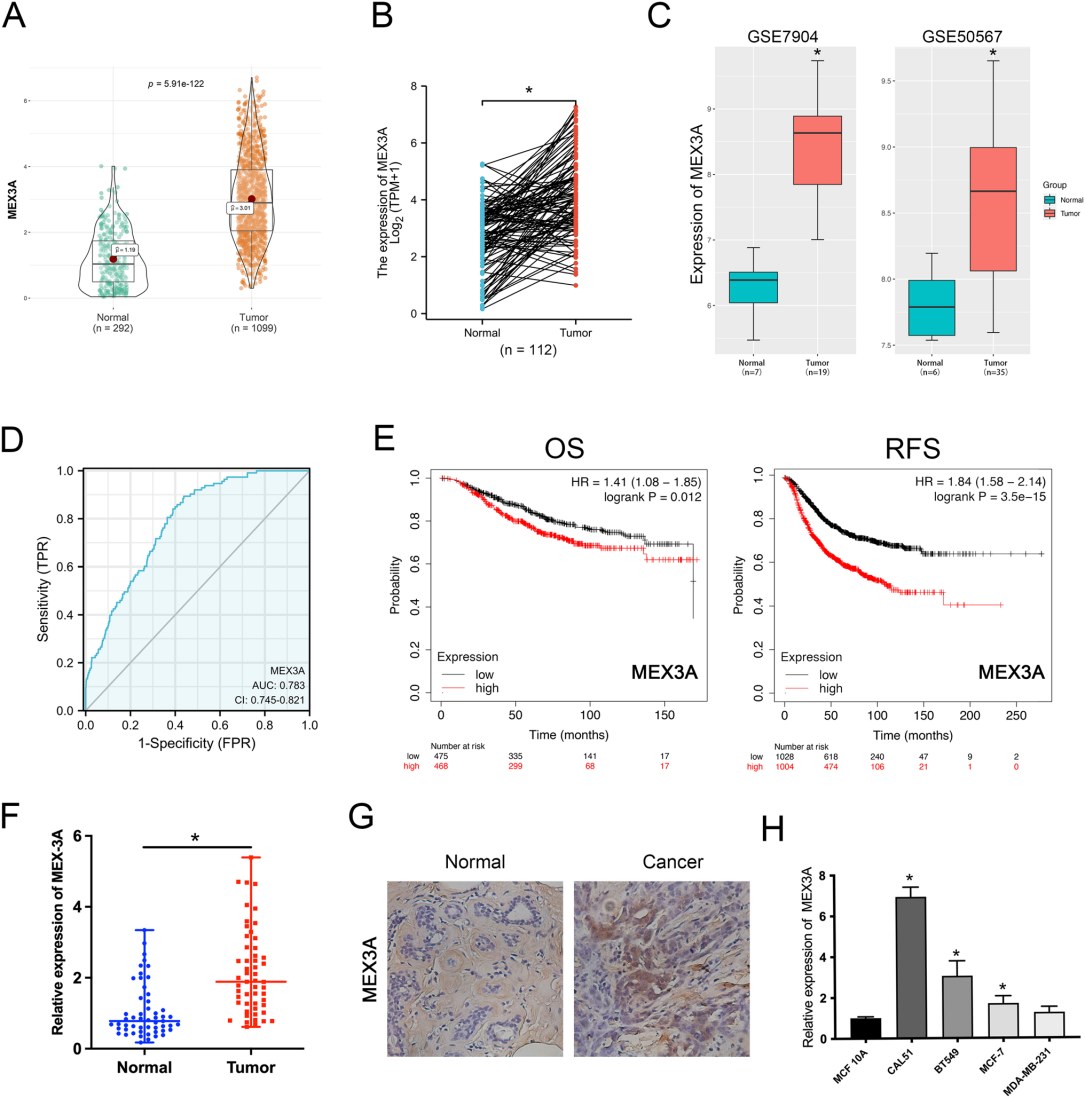
MEX3A is overexpressed in BC tissues and cell lines and predicts poor prognosis. **A**, **B** The results from the TCGA database showed that MEX3A expression was markedly upregulated in BC tissues compared to non-matched or matched normal tissues. *P < 0.05 vs. Normal group. **C** The GEO database displayed that MEX3A expression was significantly higher in BC tissues. The gene expression were log2 transformed. *P < 0.05 vs. Normal group. **D** The ROC curves analysis showed that MEX3A can be a latent discriminator between breast cancer and normal tissues. **E** The OS curves and RFS curves of BC patients generated from Kaplan–Meier plotter database. Elevated MEX3A expression was associated with an poor prognosis within patients with BC. **F** Expression levels of MEX3A in clinical samples were detected by RT-qPCR. *P < 0.05 vs. Normal group. **G** Immunostaining analysis was performed to measure the expression of MEX3A in clinical samples. **H** Expression levels of MEX3A in BC cell lines were detected by RT-qPCR. *P < 0.05 vs. control group

To explore the potential role of MEX3A in BC, we first carried out analyses of non-matched and matched tumor and normal samples in TCGA databases, and found MEX3A was significantly overexpression in BC samples (Fig. 1A, B). Then, we assessed the expression of MEX3A based on GEO databases (GSE7904, GSE50567) and determined that MEX3A is markedly up-regulated in BC tissues (Fig. 1C). Furthermore, the Cancer Cell Line Encyclopedia (CCLE) and the European Bioinformatics Institute (EMBL-EBI) bioinformatics website were also used to test the expression of MEX3A in BC cell lines, and results indicated that MEX3A was increased in most cell lines of BC (Fig. S1). The ROC curves analysis showed that MEX3A expression a potential diagnostic biomarker for BC, with AUC values of 0.783 [95% confidence interval (CI) 0.745–0.821] (Fig. 1D). MEX3A expression level was positively associated with T stage (Table 1). Afterwards, Kaplan–Meier survival analysis indicated that patients with high expression of MEX3A have a shorter OS and RFS (Fig. 1E).

**Table 1:** Correlations between MEX3A expression and clinicopathologic features of BC patients

Characteristics	Expression of MEX3A		*P* value	Method
	Low (n = 541)	High (n = 541)		
T stage, n (%)			0.006*	Chisq.test
T1	154 (14.3%)	123 (11.4%)		
T2	287 (26.6%)	342 (31.7%)		
T3	75 (6.9%)	64 (5.9%)		
T4	23 (2.1%)	12 (1.1%)		
N stage, n (%)			0.582	Chisq.test
N0	244 (22.9%)	270 (25.4%)		
N1	183 (17.2%)	175 (16.4%)		
N2	62 (5.8%)	54 (5.1%)		
N3	38 (3.6%)	38 (3.6%)		
M stage, n (%)			0.330	Chisq.test
M0	438 (47.5%)	464 (50.3%)		
M1	7 (0.8%)	13 (1.4%)		
Age, meidan (IQR)	61 (50, 70)	55 (47, 64)	< 0.001*	Wilcoxon

*T* tumor status, *N* regional lymph nodes status, *M* metastasis status, *BC* breast cancer

We further collected BC tissues and matched adjacent normal tissues (53 pairs), and RT-qPCR analysis indicated that MEX3A was remarkably overexpressed in BC tissues compared to adjacent tissues (Fig. 1F). Concurrently, immunohistochemistry (IHC) assay was performed to detect MEX3A protein expression levels. The results showed that its expression was markedly upregulated in BC tissues (Fig. 1G). MEX3A expression was higher in four BC cell lines (CAL51, BT549, MCF7, and MDA-MB231) than in a human mammary epithelial cell line (MCF-10A) (Fig. 1H). Furthermore, Confocal images displayed the distribution of MEX3A in the cytoplasm and nucleus of BT549 and CAL51 cells (Fig. S2). Altogether, these results indicate that MEX3A to be dysregulated in BC and its overexpression may be associated with the development and prognosis of BC.

### MEX3A knockdown suppresses BC cell proliferation and invasion

**Fig. 2:**
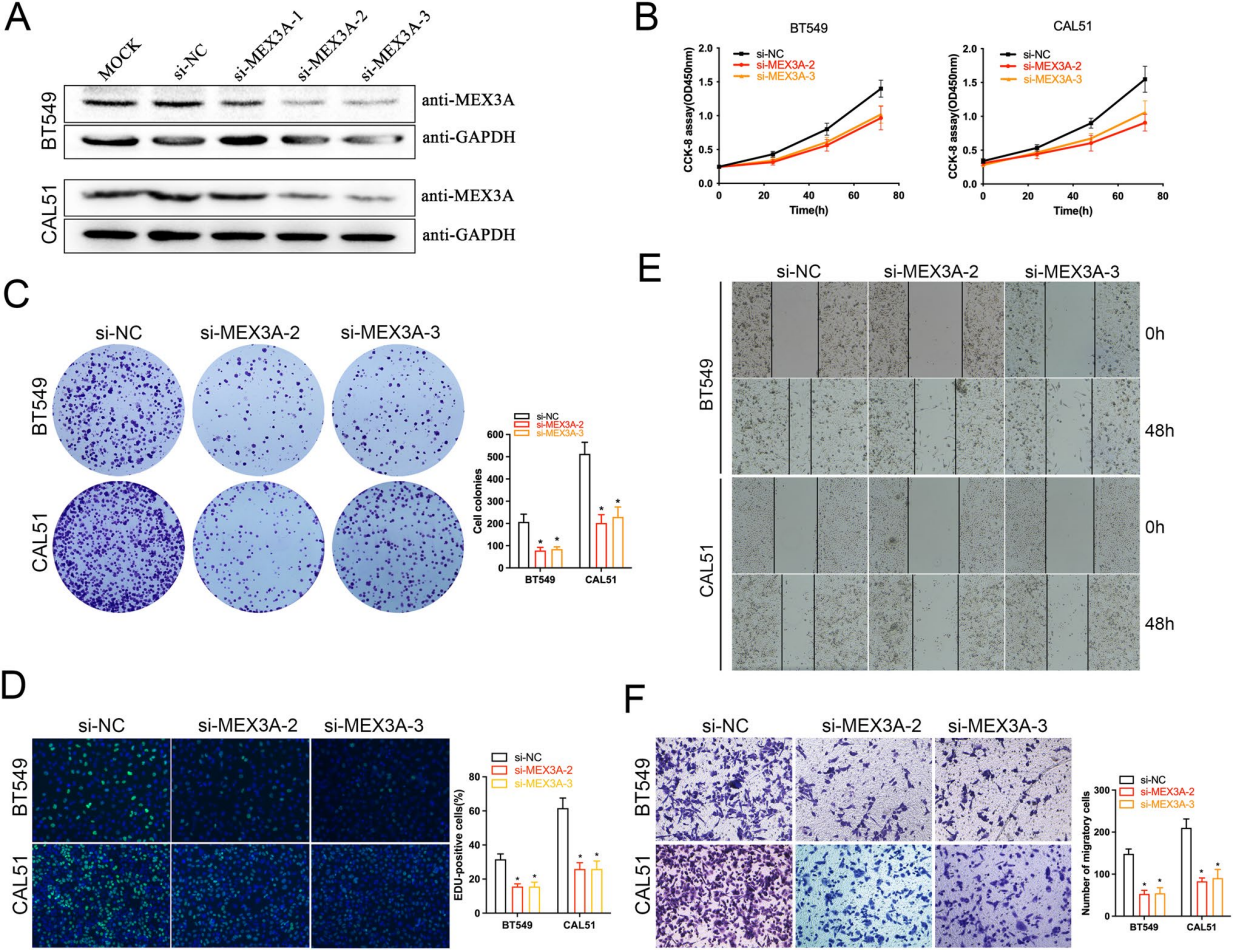
MEX3A knockdown suppresses BC cells proliferation and migration in vitro. **A** The expression of MEX3A was depleted by siRNAs in BT549 and CAL51 cells. **B**, **C** CCK-8 and colony formation assays were performed to determine the proliferation ability of BT549 and CAL-51 cells transfected with si-MEX3As. And, the colony number was counted. *P < 0.05 vs. NC group. **D** EdU assays displayed that cell proliferation was restrained by si-MEX3As in BC cells. *P < 0.05 vs. NC group. **E**, **F** Wound healing and transwell assays were performed to detect the effect of si-MEX3As on cell-migration ability. *P < 0.05 vs. NC group

To investigate the effect of MEX3A in the progression of BC, we transiently depleted MEX3A expression by siRNAs in BT549 cells and western blot analysis showed that MEX3A expression protein levels were significantly down-regulated (Fig. 2A). Then, the most effective sequence was selected to functional experiments. CCK-8 and colony formation assay showed MEX3A depletion significantly suppressed the proliferation of BT549 and CAL51 cells (P < 0.05, Fig. 2B, C). Similar results were obtained by using a EdU assay (P < 0.05, Fig. 2D). Meanwhile, wound healing and transwell assays demonstrated that MEX3A knockdown weakened migratory capacity of BT549 and CAL51 cells (P < 0.05, Fig. 2E, F). Thus, our data indicated that knockdown of MEX3A obviously suppressed the proliferation and cell invasion of BC cells in vitro.

### IGFBP4 expression is specifically regulated by MEX3A and positively correlates with favorable prognosis in BC patients

**Fig. 3:**
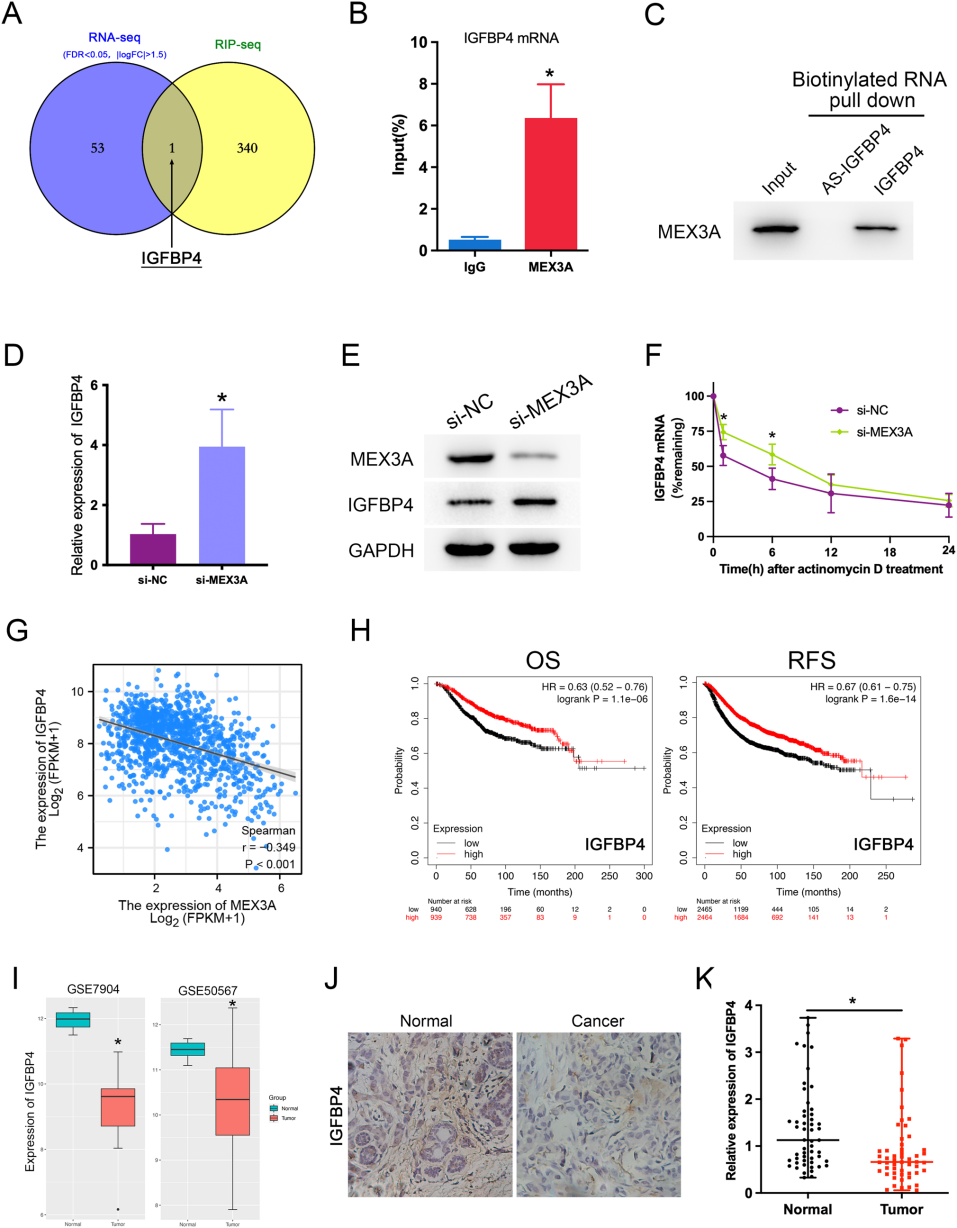
IGFBP4 is a potential target of MEX3A and correlates with favorable prognosis in BC patients. **A** Venn diagram of RNA-Seq and RIP-Seq reported MEX3A target gene. **B** RIP-qPCR analysis confirmation of MEX3A binding on IGFBP4 mRNA. The result was normalized by input group, IgG group was the negative control. *P < 0.05 vs. IgG group. **C** RNA pull-down assay and western blot analysis of the specific association of MEX3A proteins with IGFBP4 revealed that biotinylated IGFBP4 could bind to MEX3A. **D**, **E** The expression of IGFBP4 was measured by RT-qPCR and western blot in BT549 co-transfected with si-MEX3A. *P < 0.05 vs. NC group. **F** Effect of MEX3A depletion on IGFBP4 mRNA half-life was assessed in BT549 cells by actinomycin D-chase experiments. *P < 0.05 vs. NC group. **G** Spearman correlation analysis revealed a significant negative correlation between MEX3A and IGFBP4 (r = − 0.349, *P < 0.001). **H** The prognostic effect of IGFBP4 mRNA expression BC patients in from the Kaplan Meier plotter. High IGFBP4 expression was associated with favorable OS and favorable RFS in patients with BC. **I** Box-plot representation of gene expression of IGFBP4 across the BC tumors of the GSE7904 and GSE50567 dataset. *P < 0.05 vs. Normal group. **J** Immunostaining analysis was performed to measure the expression of IGFBP4 in clinical samples. **K** Expression levels of IGFBP4 in clinical samples were detected by RT-qPCR. *P < 0.05 vs. Normal group

To find out the genes associated with MEX3A regulation in BC progression, we performed crosslinked RIP-seq in BT549 cell by MEX3A antibody to detect RNA transcripts that interact with MEX3A. The result of RIP-seq analysis show that 341 mRNAs were markedly enriched by MEX3A compared to IgG control. To further evaluate the impact on the transcriptome of these interactions, we meanwhile Knockdown MEX3A and performed RNA sequencing (RNA-seq). We then compared differentially expressed genes (DEGs) from RNA-seq (|logFC|> 1.5, P value < 0.05) with Enriched genes of RIP-seq, found that IGFBP4 was the only potential target protein coding gene in both datasets (Fig. 3A, Table S1). To validate whether MEX3A might be interacted with IGFBP4 mRNA, we used RNA immunoprecipitation and quantitative PCR(RIP-qPCR) and disclosed that MEX3A could interacted directly with IGFBP4 mRNA (Fig. 3B). Next, we conducted the RNA pull down assay by biotinylated IGFBP4 mRNA in vitro and followed by a western blot as previously described. The result confirmed the ability of IGFBP4 to interact with MEX3A proteins (Fig. 3C).

Growing evidence suggests that loss of IGFBP4 in human cancer cells increases proliferation, migration, and invasion in vitro. To elucidate the regulatory effect of MEX3A on IGFBP4, we confirmed using western blot and RT-qPCR that MEX3A depletion appreciably increased IGFBP4 mRNA and protein levels (Fig. 3D, E). Furthermore, we sought to assessed the impact of MEX3A on IGFBP4 mRNA stability. Actinomycin-D chase assay showed that The half-life of IGFBP4 mRNA were significantly longer in MEX3A depleted cells at various time intervals (Fig. 3F). Next, the TCGA database was used to assess the correlation between MEX3A and IGFBP4 at the mRNA expression level. The results revealed a observable negative correlation between them (Fig. 3G). Kaplan–Meier survival analysis indicated that patients with high expression of IGFBP4 have a longer OS and RFS (Fig. 3H). Meanwhile, the GEO databases (GSE7904, GSE50567) revealed that IGFBP4 mRNA levels in BC tissues were significantly lower than non-cancerous tissues (Fig. 3I). Similarly, IHC assay indicated that IGFBP4 protein was markedly down-regulated in BC tissues (Fig. 3J). It was also shown the expression of IGFBP4 in BC tissues and matched adjacent normal tissues (Fig. 3K). Taken together, these results indicated that IGFBP4 is a direct downstream target of MEX3A and negative correlate with poor prognosis of BC patients.

### Depletion of IGFBP4 alleviated attenuated the tumor suppressive effects of MEX3A knockdown in BC cells

**Fig. 4:**
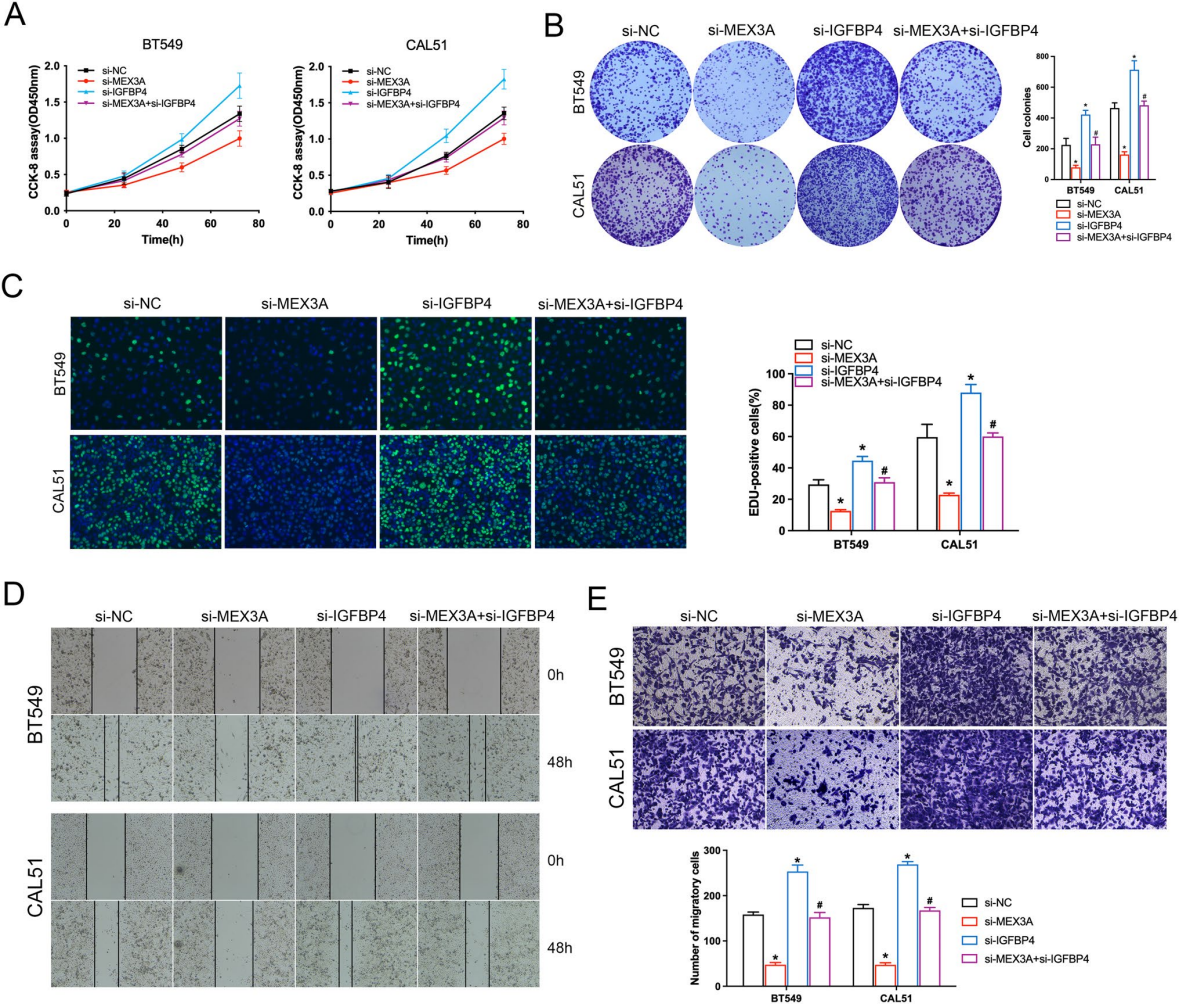
Depletion of IGFBP4 partially reverses the effect of MEX3A knockdown on BC. **A**, **B** CCK-8 and colony formation assays showed that the effects of MEX3A knockdown on cell proliferation could alleviated by IGFBP4 downregulation. *P < 0.05 vs. NC group, ^#^*P* < 0.05 vs. si-MEX3A group. **C** This pattern of results is consistent with EdU assays. *P < 0.05 vs. NC group, ^#^*P* < 0.05 vs. si-MEX3A group. **D**, **E** Wound healing and transwell assays were performed to detect the effect of si-MEX3A and si-IGFBP4 on cell-migration ability. *P < 0.05 vs. NC group, ^#^*P* < 0.05 vs. si-MEX3A group

To explore whether the IGFBP4 was critical molecule for cell proliferation restriction and invasion upon MEX3A knockdown, BC cells with MEX3A knockdown were co-transfected with si-IGFBP4. The CCK8 and colony formation assay demonstrated that silencing of both MEX3A and IGFBP4 in BC cells significantly increased cell viability compared with the si-MEX3A group (Fig. 4A, B). EdU staining also demonstrated IGFBP4 depletion to partially reverse the inhibitory effect on cell proliferation induced by si-MEX3A in BC cells (Fig. 4C). Similarly, wound healing and transwell assays demonstrated weakened invasive capacity with MEX3A knockdown, which was in part restored by si-IGFBP4 (Fig. 4D, E). These findings emphasize the novel role of IGFBP4 to inhibits tumor progression by blocking the function of MEX3A.

### Knockdown of MEX3A induces cell cycle arrest by modulation of the PI3K/AKT and MAPK signaling pathway

**Fig. 5:**
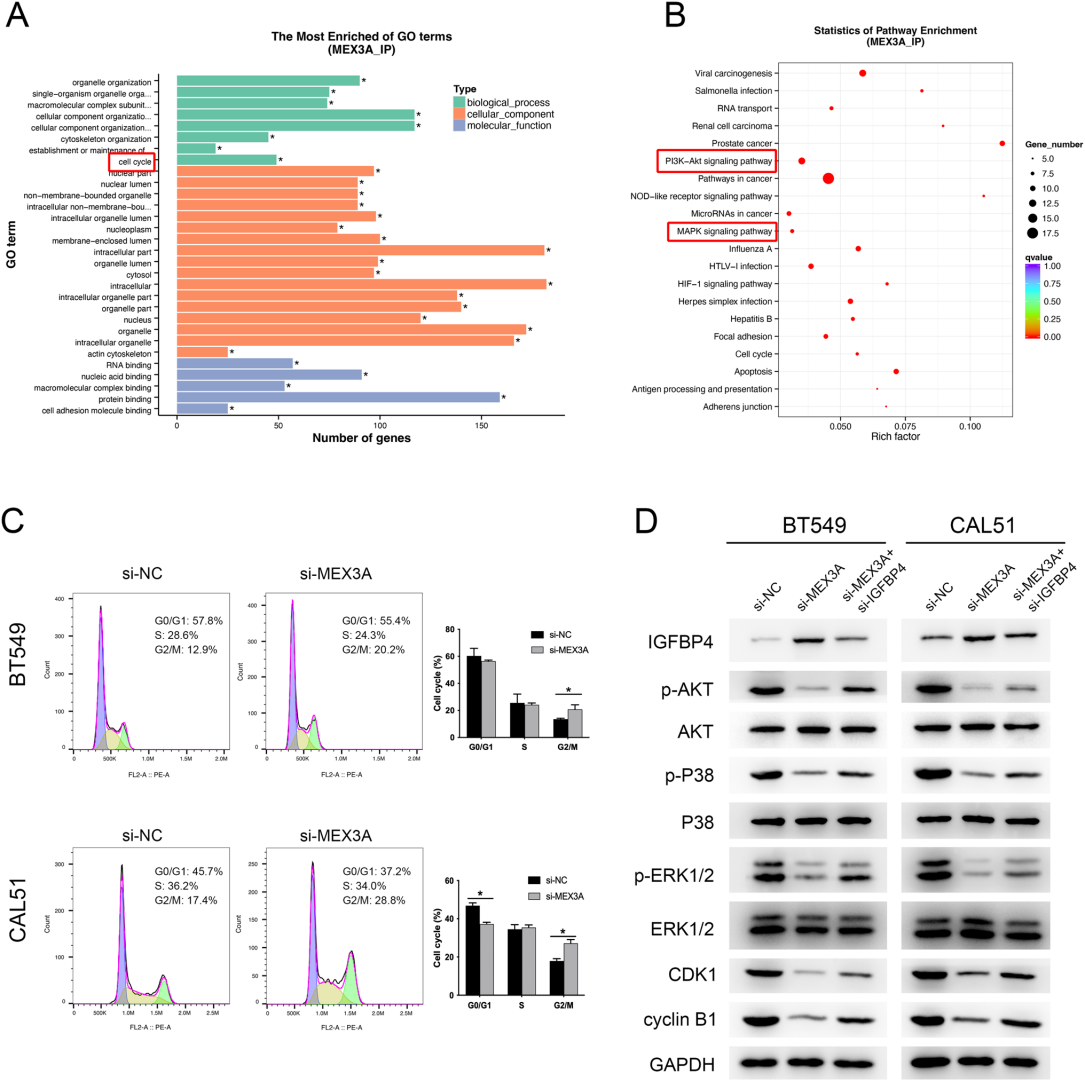
The oncogenic function of MEX3A in BC cells was dependent on cell cycle regulation. **A**, **B** GO enrichment and KEGG pathway analysis of RIP-seq peak-associated genes. **C** Effects of MEX3A knockdown on the cell cycle progression of BC cells measured by flow cytometric analysis. *P < 0.05 vs. NC group. **D** Western blot shows protein expression of PI3K/AKT, MAPK pathway and cell cycle in BC cells co-transfected with si-MEX3A and si-IGFBP4

To investigate the physiological and pathological Significance of MEX3A, GO and KEGG pathway enrichment analysis were conducted for RIP-seq peaks. These peaks were enriched for 563 GO terms and 73 KEGG pathways (P < 0.05) (Table S2). The results of GO analysis demonstrated that these peaks can be enriched in several basic biological processes(BP), including “organelle organization”, “cytoskeleton organization”, “cell cycle” (Fig. 5A). And KEGG pathway enrichment analysis showed that were mainly enriched in “Pathways in cancer”, “PI3K-Akt signaling pathway”, “MAPK signaling pathway”, “RNA transport” (Fig. 5B).

To detect whether the effect of MEX3A on BC cells proliferation was mediated via cell cycle regulation, we performed flow cytometry to examine the cell cycle distribution of BC cells. The results of cell cycle progression in BT549 and CAL51 cells using MEX3A siRNA showed that MEX3A knockdown could induce G2 phase cell cycle arrest (Fig. 5C). To further elucidate the mechanism of MEX3A in cell cycle progression, we investigate the effect of MEX3A depletion on G2/M associated cyclins and CDKs. Meanwhile, MAPK and PI3K/Akt pathway‑associated protein levels were assayed by Western blot. The analysis results revealed that knockdown of MEX3A appreciably increased expression of IGFBP4 and decreased expression of p‑AKT, p-P38, p-ERK1/2, CDK1 and cyclin B1 in BT549 and CAL51 cells compared with the si-NC group. Furthermore, downregulation of IGFBP4 expression restored the protein levels of p‑AKT, p-P38, p-ERK1/2, CDK1 and cyclin B1, partially reversing the inhibitory effect on cell progression induced by si- MEX3A in BC cells (Fig. 5D). Taken together, these results emphasize the novel role of MEX3A to regulate cell cycle targeting IGFBP4 via the PI3K/AKT and MAPK signaling pathway.

### MEX3A knockdown inhibits tumor growth of BC in vivo

**Fig. 6:**
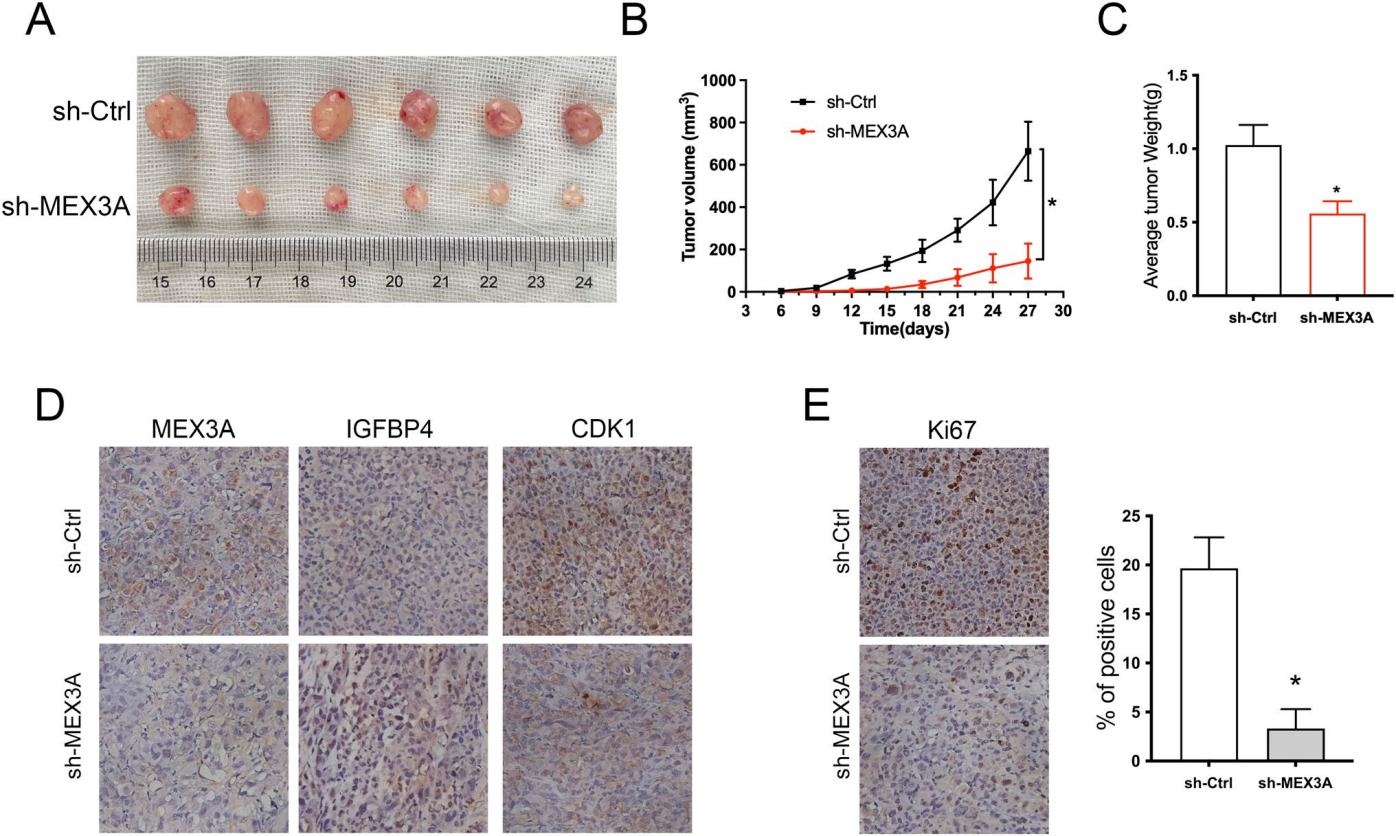
MEX3A expression positively correlates with the malignant proliferation of BC cells in vivo. **A** Effect of subcutaneous injection of BT549 cells transfected with sh-MEX3A on the tumor growth (n = 6). **B** The mean of tumor diameters for every 3 days is presented. *P < 0.05 by ANOVA. **C** Quantitative analysis of tumor weight. *P < 0.05 vs. sh-Ctrl group. **D**, **E** Immunostaining analysis was performed to measure the expression of MEX3A, IGEBP4, CDK1 and the positive rate of Ki67 in xenograft tissues

To verify the promotive effects of MEX3A on BC tumorigenesis in vivo, mice xenograft models were established through subcutaneous injection of BT549 cells that were stably transfected with sh-MEX3A. We found that the growth rate of xenograft tumors was inhibited substantially, and the tumor volumes were significantly reduced in the sh-MEX3A group (Fig. 6A, B). Meanwhile, the average tumor weight of the sh-MEX3A group was significantly lower than the sh-Ctrl group (Fig. 6C). In addition, IHC assay was demonstrated that MEX3A, and CDK1 expression were significantly lower in the sh-MEX3A group than in the sh-Ctrl group. By contrast, the expressions of IGFBP4 were elevated in the sh-MEX3A group (Fig. 6D). Meanwhile, the proliferation activity of tumors by Ki67 staining was markedly low in sh-MEX3A group (Fig. 6E). Taken together, our results suggest that MEX3A plays a vital role in the malignant behaviors of BC in vivo.

## Discussion

RNA-binding proteins (RBPs) have been shown to play a vital role in post-transcriptional regulation of mRNA [11, 12]. The abnormal expression of RBPs could affect RNA metabolism, splicing, transport, translation and other processes, and then lead to changes in the biological function of RNA in the body [13]. More than 500 RBPs have been discovered, and some of them have been proved to have tumor suppressor or carcinogenic effects [14, 15]. RBPs play regulatory roles in multiple cancer types, including lung cancer, liver cancer, gastric cancer, breast cancer and colon cancer [16–20]. Accumulating evidence illuminated that RBPs could play functional roles as tumor regulators by regulating target genes at the mRNA level [21–23]. In our study, we confirmed that MEX3A, as a novel RBP, was significantly correlated with the proliferation, metastasis and prognosis of breast cancer. And, MEX3A promote the malignant progression of breast cancer by directly targeting IGFBP4 mRNA. However, the potential mechanism of MEX3A in BC remains to be further explored.

MEX3 protein was first identified in *Caenorhabditis elegans *(*C. elegans*) in 1996 [24]. Concurrently, it was considered a translational level regulator involved in embryonic cells development. Previous studies have shown that Mutations in the MEX3 gene are lethal, Causing the embryo to improperly produce body-wall muscles from the anterior blastomere [25]. Thus, the name MEX defined for the gene is from ‘muscle excess’ [4]. Human MEX3 genes (called hMEX3A, hMEX3B, hMEX3C and hMEX3D) are located on different chromosomes and both encode phosphoproteins, expressed at diverse levels in different tissues. Human MEX3 proteins have two KH RNA-binding domains in the N-terminus, which is also present in the homology of *C. elegans* [26]. A RING finger domain with ubiquitin E3 ligase activity is present in the C-terminus, but it is absent in MEX3 of *C. elegans* [27, 28] (Fig. S3). At present, it is speculated that the function of hMEX-3 family is mainly cell self-renewal and cell differentiation, which was implicated in both cancer and stem cell biology. Previous studies have shown that MEX3A is a novel components of P bodies, which are sites of mRNA decay and storage of untranslated transcripts, indicating that MEX3A may be participated in the regulation of certain mRNA metabolism [6]. For example, Pereira et al. speculated that MEX3A involved in the post-transcriptional regulation of CDX2 by acting over its mRNA, and mediated intestinal differentiation, polarity and stemness features, implying that MEX3A may be an oncogene for colorectal cancer [29]. Jiang et al. found that downregulation of MEX3A in gastric cancer suppressed cell proliferation and migration of cancer cells, demonstrated MEX3A as a participant in development and progression of gastric cancer [8]. Moreover, Wang et al. indicated that MEX3A as a participant in the development and progression of pancreatic ductal adenocarcinoma, and that it could be potential prognosis indicator and therapeutic target [30]. In view of the above, we are urged to investigate the function and potential mechanism of MEX3A in BC and explore novel treatment strategies for BC. In this study, we found that expression of MEX3A was noticeably elevated in tumor tissues and cell lines of BC, which in accordance with the analysis of TCGA and GEO database. In addition, MEX3A expression level was positively associated with T stage and negatively correlated with survival. In clinical specimens with ER positive features, the group with high expression of MEX3A had significantly lower survival rates (*P* < 0.05). In ER negative clinical specimens, the group with high expression of MEX3A also had lower survival rates, but there was no statistical difference (*P* > 0.05) (Fig. S4). In vitro experiments show that MEX3A depletion appreciably suppresses BC cell proliferation and invasion, implying MEX3A quite likely being a tumor promoter. Similarly, MEX3A downregulation in tumor xenografts diminished significantly tumorigenicity suggested that it could be as a new therapeutic target of BC.

To explore the potential mechanism of MEX3A-mediated tumor progression in BC, RNA-seq and RIP-seq were performed to screen downstream genes in BT549 cell. Subsequently, we verified that IGFBP4 may be a downstream target of MEX3A in the regulation of BC. Specifically, IGFBP4 was downregulated in BC tissues, which was significantly negatively correlated with MEX3A. Kaplan–Meier survival analysis of TCGA database indicated that patients with high expression of IGFBP4 have a favorable prognosis. Meanwhile, the expression of MEX3A and IGFBP4 correlated with triple-negative breast cancer (TNBC) of the lack of estrogen and progesterone receptors and HER2 (Fig. S4). Increasing evidence illuminated that IGFBP4 plays a prominent role in different malignant tumors [31]. In hepatocellular carcinoma, IGFBP4 as a novel tumor suppressor potently inhibits cell proliferation, invasion, and diminishes xenograft tumor growth in mice by the EZH2 and AKT pathways [32]. In lung cancer, IGFBP4 serum protein levels are appreciably elevated in tumor samples, and it is adversely associated with the long-term prognosis of patients [33, 34]. Durai et al. verified that abnormally low Expression of IGFBP4 is a critical reason for excessive cell proliferation in colorectal cancer [35]. However, the involvement of IGFBP4 in breast cancer progression has not been previously reported. In the present study, we performed loss-of-function experiments and found that IGFBP4 effectually reversed tumor inhibition caused by MEX3A depletion. Meanwhile, Overexpression of MEX3A in BC cells significantly increases cell proliferation and migration, while co-overexpression of IGFBP4 partially reverses the above results (Fig. S5). Subsequently, RIP and RNA pull-down assays confirmed the ability of MEX3A proteins to directly interact with IGFBP4 mRNA. Furthermore, GO and KEGG pathway enrichment analysis separately revealed that MEX3A had important effects on genes that are mainly related to cell cycle, PI3K-Akt and MAPK pathway, and IGFBP4 could reverses the effect of that, which were further verified by Western Blot.

In conclusion, our study demonstrated that MEX3A, a novel carcinogenic RBP in BC, could promote tumor cell progression through mediated IGFBP4 post-transcriptional metabolism, ultimately affecting survival in BC patients. The newly discovered crucial roles of MEX3A and IGFBP4 in tumor progression provide theoretical basis of pathogenesis and potential novel strategy for the treatment of BC.

## Materials and methods

### Bioinformatics analysis

We first investigated the expression level of MEX3A between BC and normal samples at The Cancer Genome Atlas (TCGA) and Genotype-Tissue Expression (GTEx) databases. Gene expression data were visualized by the “Vioplot” or “Paired Plot” function of the R language. With the goal of improving the reliability of the results, Microarray datasets (GSE7904, GSE50567) from the GEO database were used to test MEX3A differential expression for BC. The area under the curve (AUC) of the receiver operating characteristic (ROC) curve was evaluated to assess the discriminatory capability of MEX3A for Breast cancer. The Kaplan–Meier plotter was used to assess the prognostic value of MEX3A expression in patients with BC. Processing of the data was according to the guidelines of these databases.

### Tissue samples and cell culture

All BC and adjacent noncancerous breast tissues in this study were collected from Cancer Hospital of the University of Chinese Academy of Sciences (Zhejiang Cancer Hospital). The tissues were immediately snap frozen in liquid nitrogen until RNA extraction. Patients did not receive chemotherapy or radiotherapy and signed informed consent. This study was approved by the Medical Ethics Committee of Cancer Hospital of the University of Chinese Academy of Sciences. The BC cell lines (CAL51, BT549, MCF7, and MDA-MB231) and the human mammary epithelial cell line (MCF-10A) were purchased from ATCC (Manassas, VA, USA) and cultured according to the manufacturer’s instructions.

### Immunohistochemistry

Immunohistochemistry (IHC) was performed according to a standard streptavidin–biotin-peroxidase complex method. In brief, tissue samples were embedded in paraffin and cut to a thickness of 5 μm. Sections were stained with primary antibodies reactive. Then, washed and incubated with horseradish peroxidase-conjugated secondary antibodies. Staining results were visualized by sequential incubations of sections with the DAB system, and the nucleus was counterstained with hematoxylin. Images of representative fields were obtained using the Zeiss microscope.

### RT-qPCR and western blot assays

Total RNA was extracted from BC tissues and cells by using TRIzol reagent. The quantitative analysis of RNAs was performed by NanoDrop 2000 Spectrophotometer. cDNA synthesis was done with a PrimeScript RT reagent Kit under the guidance of the manufacturer’s instructions. RT-qPCR was performed using the TB Green Premix Ex Taq kit. The primers used were as follows: MEX3A F: CATGGCAGCAGTAGCAGTAACAATC, MEX3A R: CAAGGACAGTGTTTCCACTCCA, IGFBP4 F: GCAAGATGAAGGTCAATGGG, IGFBP4 R: GATGTAGAGGTCCTCGTGG, β-actin F: TGGCACCCAGCACAATGAA, β-actin R: CTAAGTCATAGTCCGCCTAG AAGCA. Relative gene expression was normalized to β-actin based on the 2^−ΔΔCt^ method.

Cells were harvested and lysed in RIPA buffer and the total protein was extracted. Protein concentrations were detected using the BCA Protein Assay Kit. Total protein (40 μg) was separated on an SDS-PAGE gel and transferred to a PVDF membrane (Millipore, USA), and immunoblotted with the respective primary and secondary antibodies. Protein signals were detected with ECL reagents.

### Transfection of cells

Three individual MEX3A (si-MEX3A-1,-2,-3), IGFBP4 (si-IGFBP4), and negative control(si-NC) small interfering RNA (siRNA) were designed and purchased from GenePharma Co., Ltd. (Shanghai, China). The most efficient sequence was packaged as lentiviruses (sh-MEX3A) by GenePharma. Cell transfection was performed using Lipofectamine 2000 (Invitrogen) as described in the manufacturer’s instructions.

### CCK-8 assay

Cell viability was assessed by Cell Counting Kit-8 (CCK-8) according to the manufacturer’s protocol. In brief, BC cells were seeded at a density of 1 × 103 cells/well in 96-well plates. After incubation for 0, 24, 48, and 72 h, 10 μL of CCK-8 solution (Dojindo, Japan) was added to each well. Then, the absorbance was determined by using a microplate reader set at a wavelength of 450 nm. The experiments were repeated in triplicate, independently.

### EdU proliferation analysis

An ethynyl deoxyuridine (EdU) kit (Invitrogen) was used to assay cell proliferation based on the manufacturer’s instructions. Briefly, BC cells were seeded in 24-well plates for transfection with siRNA oligonucleotide. After incubation at 37 °C and 5% CO_2_ for 48 h, cells were incubated with 50 mM EdU for 2 h before fixation, permeabilization and EdU staining. Nucleicacids in all cells were stained with DAPI(Sigma) at a concentration of 1 mg/mL. The proportion of cells that incorporated EdU were observed by fluorescence microscopy with results quantified by counting at least three random fields.

### Colony formation assay

BC cells transduced with siRNA oligonucleotide were placed into six-well plates and cultured in a humidified incubator for 2 weeks. The medium was replaced every 3 days. After 2 weeks, Cell colonies were washed twice with PBS and fixed with cold paraformaldehyde for 20 min followed by a staining with 0.1% crystal violet at room temperature for 1 h. Triplicate wells were measured for each treatment group and visible colonies were counted for quantification of results.

### Wound-healing assay

Transfected cells were seeded in six-well plates and incubated at 37 °C overnight. The next day when cells reached full confluent, the monolayer was manually scraped to create wound areas using a pipette tip. The cultures were continued at 37 °C, and observed and photographed at 0 and 48 h after wounding.

### Transwell assays

Transfected cells in FBS-free medium were seeded into the upper chamber that had been pre-coated with Matrigel. In the lower chambers, medium containing 10% FBS was added as a chemoattractant. Following 24 h incubation, non-migrated cells in the upper chamber were removed with cotton swabs. The migrated cells on the lower chamber surface were fixed with paraformaldehyde and stained with 0.1% crystal violet, and enumerated by light microscopy at three different fields of view.

### RIP sequencing and RIP-qPCR

Approximately 20 million BT549 cells were collected and washed with PBS. RNA-immunoprecipitation (RIP) was performed using a Magna RIP Kit (Millipore) in accordance with the manufacturer's protocol. Briefly, Cell were lysed for 60 min on ice in RIP Lysis Buffer containing protease inhibitor and RNase inhibitor. Then, 50 μL of magnetic beads were coupled to anti-MEX3A or anti-rabbit-IgG antibodies. Cell lysates were incubated with the pellet (beads-antibody complex) overnight at 4 °C. Immunoprecipitated RNA was purified with phenol chloroform from the pellet and collected for RNA library construction and finally sequenced on an Illumina HiSeq Sequencer according to the manufacturer’s instructions at Novogene Technology (Beijing, China) as described in detail in Table S1. For RIP-qPCR, the immunoprecipitate were treated with proteinase K and then used for RT-qPCR.

### RNA sequencing

Total RNA was isolated from MEX3A knockdown or control BT549 cells by using Trizol reagent. The high‐throughput sequencing and analyses for mRNA were carried out by Novogene Technology. Analyzed data are available in Table S1.

### RNA pull-down assay

IGFBP4 or antisense RNA was transcribed in vitro from the pcDNA3.1 vector by TranscriptAid T7 High Yield Transcription Kit (Thermo Scientific). The transcribed IGFBP4 was then purified using a PureLink RNA Mini Kit (Thermo Scientific). Then, the transcripts were biotin labeled with the Biotin 3′ End DNA Labeling Kit (Thermo Scientific). Whole-cell lysate from BT549 cell was then incubated with biotinylated RNA for 1 h at room temperature. The RNA–protein complex was isolated with streptavidin magnetic beads followed by the binding protein isolated from it for further Western blot analysis.

### RNA stability assay

Actinomycin D treatment assay was performed for detecting IGFBP4 mRNA stability. Transfected cells cultured after 48 h in vitro were treated with actinomycin D at a concentration at 5 µM for the indicated time. RNA-extractions were performed at 0 h, 1 h, 6 h, 12 h, and 24 h and used for RT-qPCR analysis. Levels of β-actin mRNA are expressed as a percentage of the β-actin mRNA remaining at each experimental time compared with time zero. IGFBP4 mRNA were calculated as the ratio of the level of β-actin mRNA.

### Cell cycle analysis

Transfected cells were seeded in 6-well plates, and then collected and washed with PBS two times and fixed with 70% ethanol at 4 °C overnight. The fixed cells were incubated with propidium iodide for 30 min. Finally, the cells were assayed by flow cytometry (BD, USA). Quantitative cell cycle analysis was conducted using FlowJo software.

### Tumor Xenograft model

Female BALB/c nude mice (5 weeks old) were purchased from Shanghai SLAC Laboratory Animal Co., Ltd. (Shanghai, China). BT549 cells transfected with sh-MEX3A resuspended in PBS were injected directly into the mammary fat pad. The tumor growth was monitored weekly with a vernier caliper and calculated by using the formula: volume = (length × width^2^)/2. All the mice were performed euthanasia 5 weeks after inoculation, and their tumor xenografts were removed, weighed, imaged, and prepared for immunohistochemistry according to the manufacturer's protocol.

### Statistical analysis

All data were analyzed with GraphPad Prism 9.0 software. A Student’s t-test was used to analyze two group comparisons. The associations among MEX3A and clinical characteristics of BC patients were assessed by the chi-square test. Statistical correlations among MEX3A and IGFBP4 expression were evaluated by Spearman’s analysis. Differences were considered statistically significant at P < 0.05.

## Supplementary Information

Below is the link to the electronic supplementary material.

Supplementary file1 **Figure S1**. The Expression of MEX3A in BC Cell Lines (CCLE and EMBL-EBI). (A) The expression of MEX3A in BC cell lines, analyzing by CCLE. (B) The expression of MEX3A in BC cell lines, analyzed by EMBL-EBI. (TIF 51613 KB)

Supplementary file2 **Figure S2**. Subcellular localization of MEX3A proteins. Immunofluorescence for MEX3A were stained with anti-MEX3A and analysed by confocal microscopy. Scale bar: 50 μm. (TIF 72127 KB)

Supplementary file3 **Figure S3**. Schematic Representation of the Structural Domains of MEX3 genes. The figure shows a simplistic representation of the different structural domains of MEX3 genes, including KH domains and zinc finger domains. The amino acid length of MEX3 genes are also mentioned. (TIF 19891 KB)

Supplementary file4 **Figure S4**. Comparison of ER/PR/HER2 status with the expression of MEX3A and IGFBP4. (A) The OS curves of BC patients generated from Kaplan-Meier plotter database. Elevated MEX3A expression was associated with an poor prognosis within ER-postive patients (*P* < 0.05). (B) The OS curves of BC patients generated from Kaplan-Meier plotter database. The group with high expression of MEX3A also had lower survival rates, but there was no statistical difference (*P* > 0.05). (C) The correlation of expression of MEX3A and IGFBP4 with ER status. (D) The correlation of expression of MEX3A and IGFBP4 with PR status. (E) The correlation of expression of MEX3A and IGFBP4 with HER2 status. (TIF 34984 KB)

Supplementary file5 **Figure S5**. Upregulation of IGFBP4 attenuates the promoting role of MEX3A overexpression on BC. (A, B) CCK-8 assays showed that the effects of MEX3A overexpression on cell proliferation could alleviated by IGFBP4 Upregulation. (C) Transwell assays were performed to detect the effect of OE-MEX3A and OE-IGFBP4 on cell-migration ability. ∗*P* < 0.05 vs. Vector group, ^#^*P* < 0.05 vs. OE-MEX3A group. (TIF 19781 KB)

Supplementary file6 (XLSX 8065 KB) **Table S1.** RIP-seq and RNA-seq data.

Supplementary file7 (XLSX 521 KB) **Table S2.** GO terms and KEGG pathways.

## Data Availability

The authors declare that all data supporting the findings of this study are available within the article.
