# Supplementary figures and images for: The RNA binding protein MEX3A promotes tumor progression of breast cancer by post-transcriptional regulation of IGFBP4
Source: Breast Cancer Res Treat. 2023 Jul 11;201(3):353–66. doi: 10.1007/s10549-023-07028-5 (PMC10460732; doi:10.1007/s10549-023-07028-5)

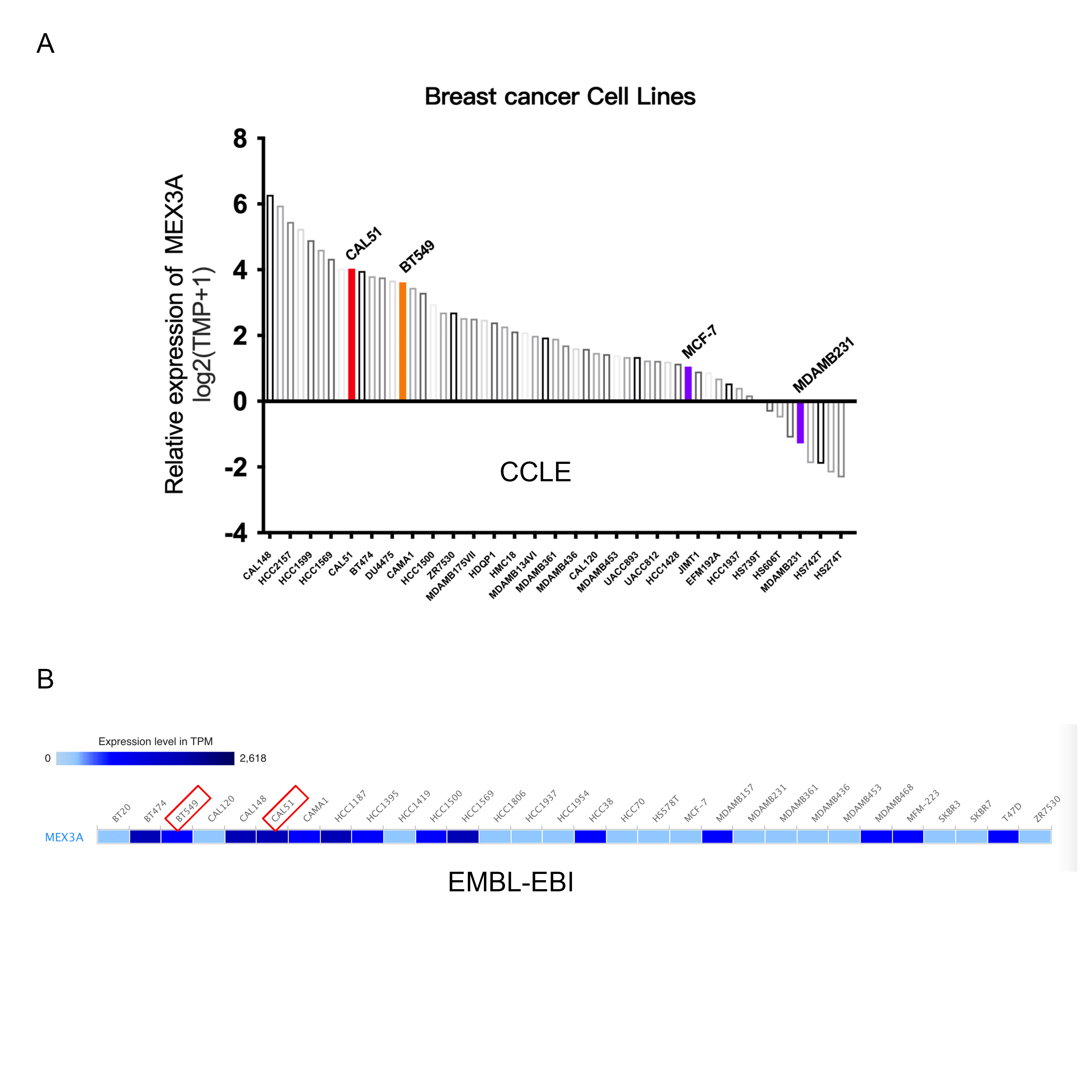

Supplement: Supplementary file 1 — Supplementary file1 Figure S1. The Expression of MEX3A in BC Cell Lines (CCLE and EMBL-EBI). (A) The expression of MEX3A in BC cell lines, analyzing by CCLE. (B) The expression of MEX3A in BC cell lines, analyzed by EMBL-EBI. (TIF 51613 KB) [file 10549_2023_7028_MOESM1_ESM.tif]

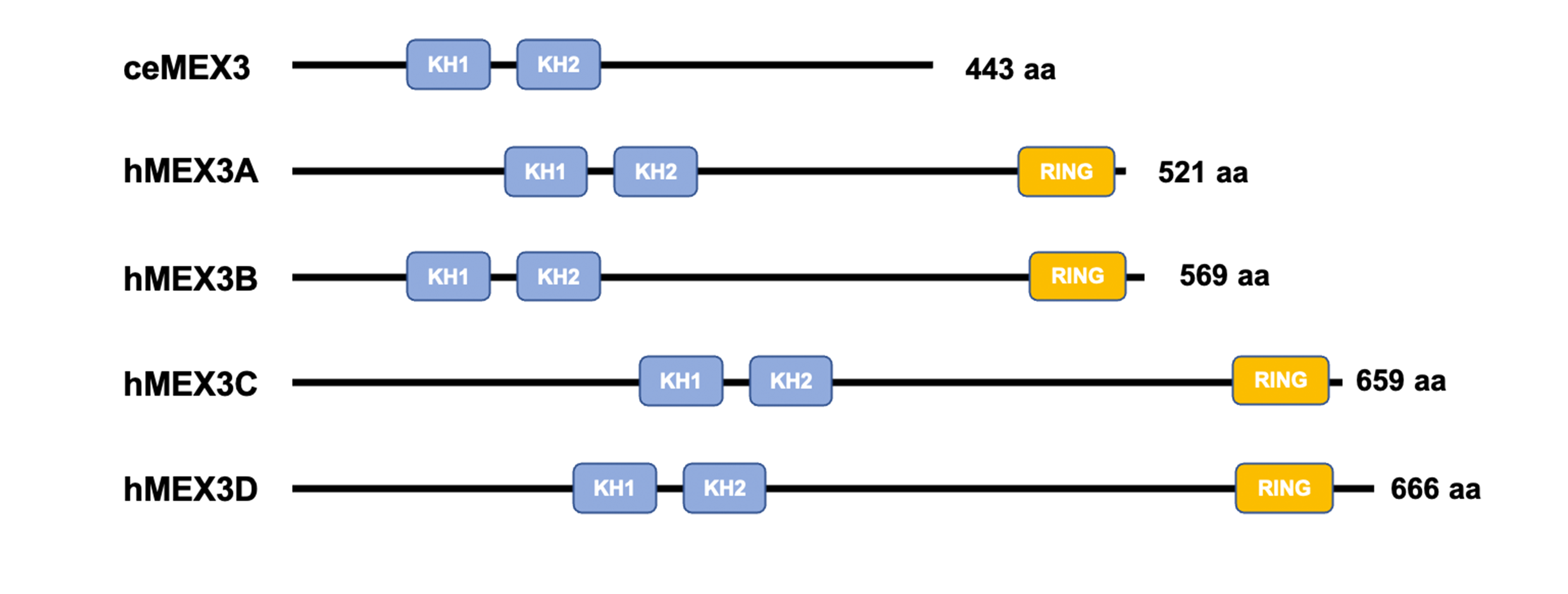

Supplement: Supplementary file 3 — Supplementary file3 Figure S3. Schematic Representation of the Structural Domains of MEX3 genes. The figure shows a simplistic representation of the different structural domains of MEX3 genes, including KH domains and zinc finger domains. The amino acid length of MEX3 genes are also mentioned. (TIF 19891 KB) [file 10549_2023_7028_MOESM3_ESM.tif]

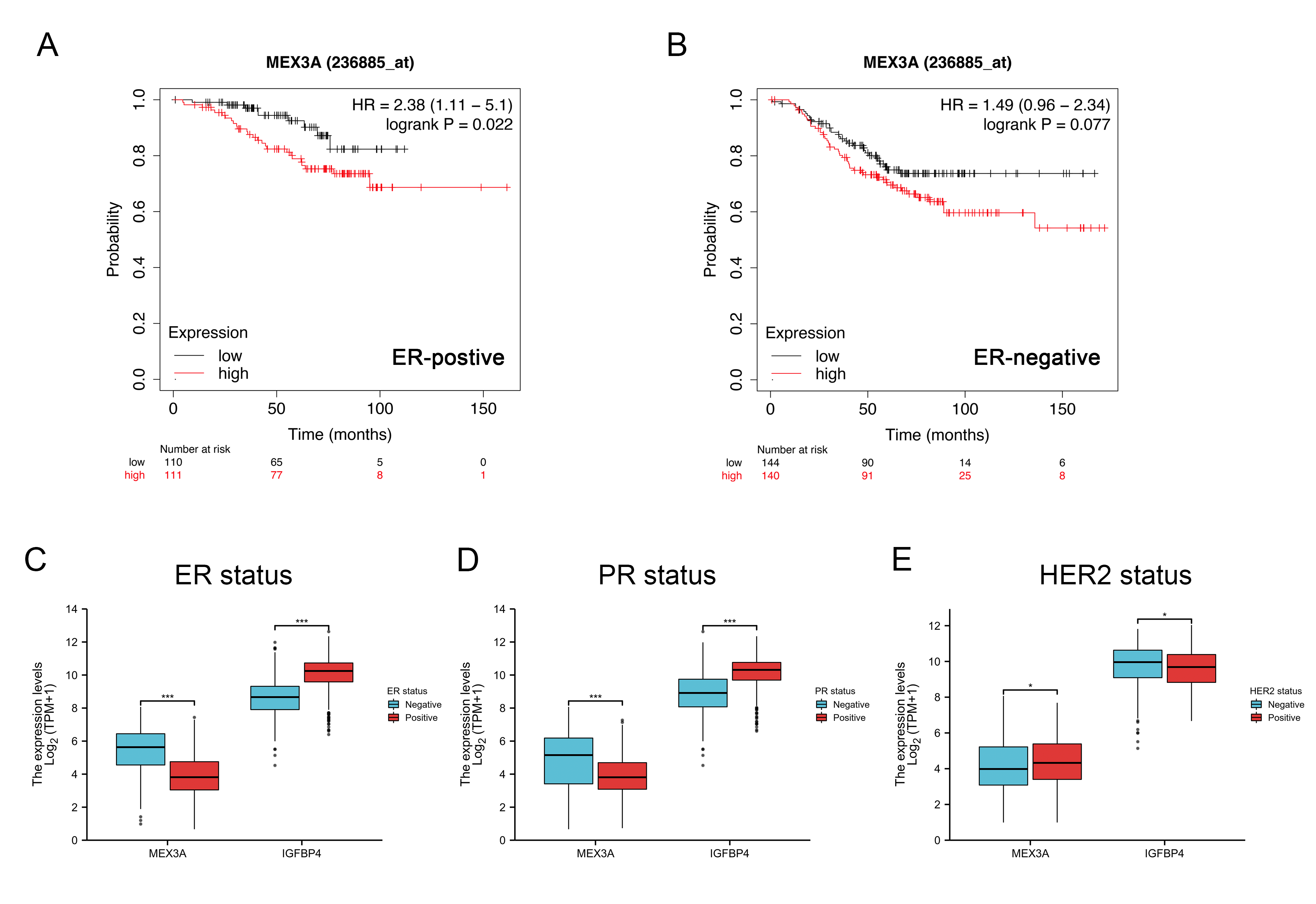

Supplement: Supplementary file 4 — Supplementary file4 Figure S4. Comparison of ER/PR/HER2 status with the expression of MEX3A and IGFBP4. (A) The OS curves of BC patients generated from Kaplan-Meier plotter database. Elevated MEX3A expression was associated with an poor prognosis within ER-postive patients (P < 0.05). (B) The OS curves of BC patients generated from Kaplan-Meier plotter database. The group with high expression of MEX3A also had lower survival rates, but there was no statistical difference (P > 0.05). (C) The correlation of expression of MEX3A and IGFBP4 with ER status. (D) The correlation of expression of MEX3A and IGFBP4 with PR status. (E) The correlation of expression of MEX3A and IGFBP4 with HER2 status. (TIF 34984 KB) [file 10549_2023_7028_MOESM4_ESM.tif]

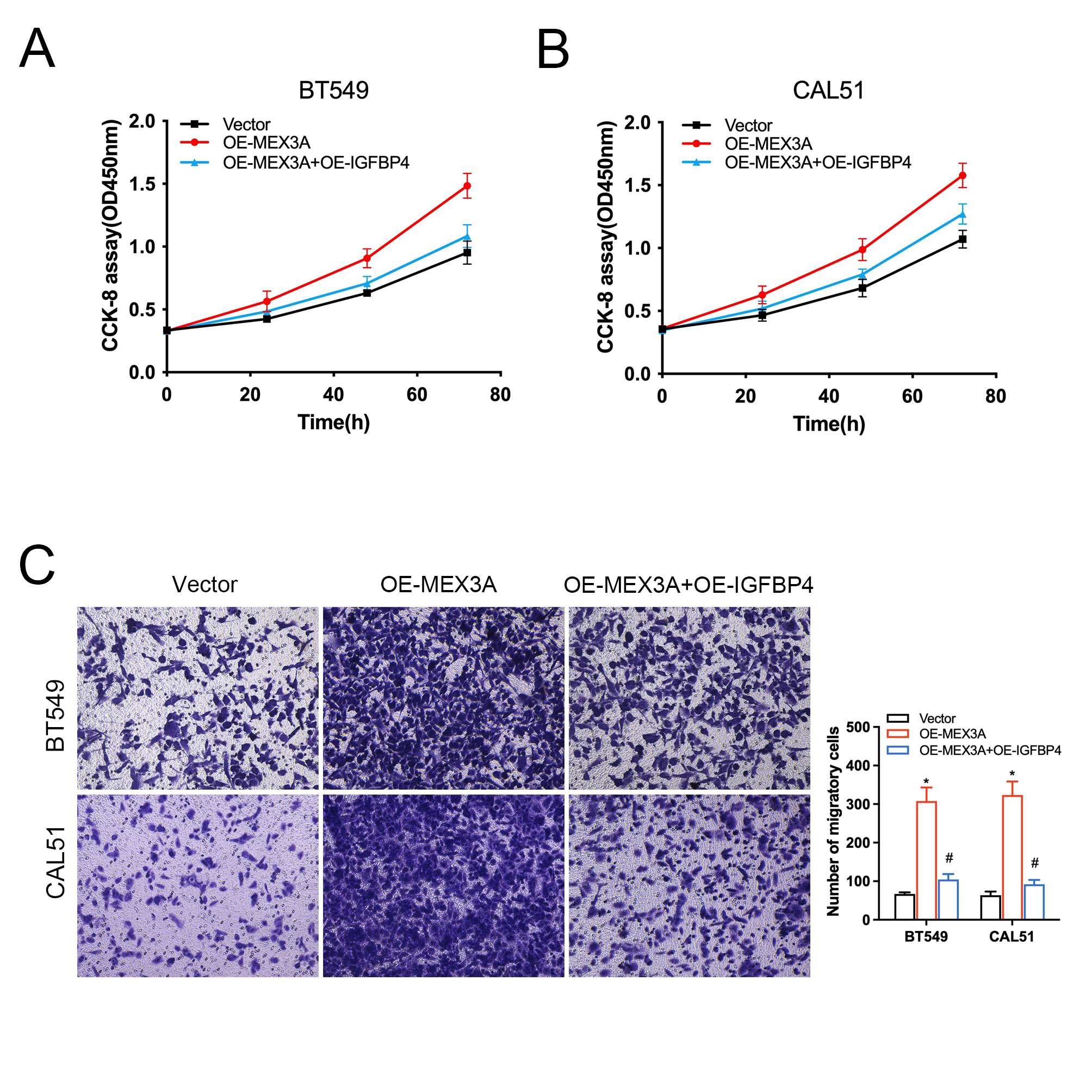

Supplement: Supplementary file 5 — Supplementary file5 Figure S5. Upregulation of IGFBP4 attenuates the promoting role of MEX3A overexpression on BC. (A, B) CCK-8 assays showed that the effects of MEX3A overexpression on cell proliferation could alleviated by IGFBP4 Upregulation. (C) Transwell assays were performed to detect the effect of OE-MEX3A and OE-IGFBP4 on cell-migration ability. ∗P < 0.05 vs. Vector group, #P < 0.05 vs. OE-MEX3A group. (TIF 19781 KB) [file 10549_2023_7028_MOESM5_ESM.tif]
